# A multidisciplinary pediatric oncofertility team improves fertility preservation and counseling across 7 years

**DOI:** 10.1002/cnr2.1753

**Published:** 2022-11-08

**Authors:** John A. Ligon, Masanori Hayashi, Devon Ciampa, Cara Kramer, Alfredo Guastella, Robert J. Fuchs, Amin S. Herati, Mindy S. Christianson, Allen R. Chen

**Affiliations:** ^1^ Sidney Kimmel Comprehensive Cancer Center Johns Hopkins University (JHU) Baltimore Maryland USA; ^2^ Department of Pediatrics, Division of Hematology/Oncology University of Florida Gainesville Florida USA; ^3^ Department of Pediatrics—Hematology/oncology and Bone Marrow Transplantation University of Colorado Aurora Colorado USA; ^4^ James Buchanan Brady Urological Institute and Department of Urology JHU Baltimore Maryland USA; ^5^ Division of Reproductive Endocrinology and Infertility JHU Baltimore Maryland USA

**Keywords:** fertility counseling, fertility preservation, oncofertility, pediatric oncology, quality improvement

## Abstract

**Background:**

Oncofertility is a developing field of increasing importance, particularly in pediatric oncology, where most patients are likely to survive long‐term and have not yet had the opportunity to have children.

**Aims:**

We performed a quality improvement initiative to increase our rates of fertility preservation counseling and referral through the implementation of a pediatric oncofertility team, and we report outcomes 7 years following implementation of our initiative.

**Methods and results:**

We compare our baseline oncofertility survey to 44 post‐intervention survey respondents and electronic medical record documentation for 149 patients treated in 2019. Ninety‐five percent of post‐intervention survey respondents recalled fertility counseling (baseline 70%, *p* = .004) and 89.3% were appropriately referred for fertility preservation (baseline 50%, *p* = .017). Counseling was documented in 60.4% of charts; 81% of patients analyzed by chart review were appropriately referred for fertility preservation. Fertility preservation outcomes differed by sex assigned at birth.

**Conclusion:**

Creation of an oncofertility team produced improvements in fertility counseling and fertility preservation referral across an extended period of time.

## 1 INTRODUCTION

Therapy‐related infertility following cancer treatment profoundly impacts quality of life, particularly for pediatric patients.^1^, ^2^, ^3^, ^4^, ^5^ While the American Society of Clinical Oncology and the American Academy of Pediatrics promote fertility risk counseling and fertility preservation (FP) referral,^6^, ^7^ the rate of these actions remains suboptimal.^8^, ^9^ Urgency to initiate therapy quickly and discomfort with discussing fertility contribute to suboptimal outcomes.^10^ While developing an oncofertility program has improved patient satisfaction and FP referral in adults,^11^, ^12^ continued improvement is needed. Many Children's Oncology Group member institutions report the creation of a designated FP individual or team and that this has improved access to FP procedures,^13^ and some individual centers note an increase in FP counseling and referral in electronic medical record (EMR) documentation following the implementation of such a designated FP team.^14^, ^15^, ^16^ Specific defined metrics to measure “success” of an FP team, such as rate of FP counseling and FP referral for at‐risk patients, are needed to measure the effectiveness of these interventions. Ideally, all patients should receive counseling on the risk of infertility prior to starting genotoxic therapy with curative intent.

A baseline survey of fertility practices at Johns Hopkins Hospital (JHH) pediatric oncology clinic revealed opportunity for improvement.^17^ We undertook a quality improvement initiative to institute a pediatric oncofertility team to support standardized FP counseling and referral. We have analyzed EMR documentation in combination with patient‐level survey responses to assess the impact of this intervention and to identify opportunities for further improvement.

## 2 METHODS

### 2.1 Baseline survey

A simple survey of 20 patients at the Johns Hopkins pediatric oncology clinic regarding receipt of FP counseling and referral was collected in 2012 and was previously presented at the 2013 ASCO Quality Care Symposium.^17^

### 2.2 Pediatric oncofertility team

A QI initiative established a multidisciplinary pediatric oncofertility team comprising physicians, social workers, and nurses in 2013.^17^ Systematic interventions were implemented, including: (1) Establishment and maintenance of a comprehensive standard practice for fertility preservation and counseling (last updated in 2018) which is published internally in an online policy library; (2) Annual lectures for faculty and fellows, and training for nurses undergoing orientation; (3) Clearly outlining the referral process for fertility preservation procedures; (4) Increased collaboration with colleagues in reproductive endocrinology and urology to ensure that our division remained aware of available fertility preservation methods, including oocyte cryopreservation, ovarian tissue cryopreservation (OTC, available on a research basis initially and then available as our standard of care beginning in 2017), sperm cryopreservation, and invasive methods of sperm retrieval (including epididymal sperm aspiration, electroejaculation, and testicular sperm extraction); and (5) On‐call availability of a physician on the oncofertility team to answer questions regarding fertility. Our standardized policy was published in our internal online policy database and accessible by anyone from any discipline providing care to pediatric oncology patients at Johns Hopkins (Supplemental methods S1). Criteria based on sex assigned at birth categorizing infertility risk were developed^18^, ^19^, ^20^ (Supplemental methods S2). Counseling and referral algorithms were created (Supplemental Figure S1). While we recognize the conflicting data regarding the efficacy of gonadotropin‐releasing hormone (GnRH) agonists for female fertility preservation,^21^, ^22^ given their other potential benefits including decreased breakthrough bleeding in the setting of thrombocytopenia,^23^, ^24^ we consider their use in females who are at least Tanner Stage 2. Our goal was to provide guidance to enable the primary treating team who have the strongest relationship with the patient to initiate FP counseling and referrals, with the intent that all patients would receive FP counseling, and if appropriate, FP referral, prior to the initiation of any new line of treatment (i.e., at diagnosis or progression/relapse).

### 2.3 Post‐intervention survey and chart review

A QI project assessment (approved by Johns Hopkins Medicine Institutional Review Board, IRB# 00243941) survey was conducted with a post‐intervention survey in 2020 (Supplemental methods S3). This survey included the questions from the baseline survey with added detail, including questions for us to better assess patient perceptions and recollections of FP counseling for the design of future QI initiatives. Parents answered by proxy for minor patients. Only responses from patients who started therapy in the years from 2014 to 2020 were analyzed (Supplemental Figure S2A). The patient's EMR (EPIC, Verona WI) was reviewed and compared against survey responses.

Recognizing that our post‐intervention survey respondents may not be representative of our total pediatric oncology patient population receiving gonadotoxic therapy, we also performed an EMR review of patients who started therapy in 2019 to evaluate recent oncofertility outcomes (Supplemental Figure S2B). Because all facility‐administered chemotherapy is ordered using treatment plans created in our electronic medical record (EPIC, Verona WI), we utilized its reporting functions to identify all treatment plans created in 2019 for patients under age 25. We then identified unique patients and excluded patients who: (1) Were not treated by the pediatric oncology division (young adults ages 18 to 25 could be managed by pediatric oncology or adult medical oncology); (2) Had received recent gonadotoxic therapy prior to the treatment plan initiated in 2019 without sufficient time for recovery (defined as less than 6 months off therapy); (3) Did not actually start therapy in 2019 (treatment plan was created, but therapy was not delivered); or (4) received only non‐cytotoxic therapy that would not be expected to impact fertility (Supplemental Figure S2B). The medical records were reviewed for: (1) Age; (2) Diagnostic category (as outlined above); (3) Documentation of fertility risk discussion; (4) Documentation of whether the patient was referred for fertility preservation procedures; (5) Documentation of the outcome of referral for fertility preservation; and (6) Whether the patient would have fertility preservation recommended based on our algorithms (Supplemental Figure S1). Notes and the patient's problem list were reviewed for the above documentation. Additional reasons why a patient would not have fertility preservation recommended included: (1) Anatomic barriers to OTC when urgent therapy was required; (2) Prior chemotherapy at cumulative doses considered to cause infertility already; and (3) Transgender patient.

### 2.4 Measures

Receipt of counseling and referral of patients meeting criteria were selected as primary outcomes. Documentation of counseling and referral were the outcomes in the chart‐review cohort. Administration of a GnRH agonist was not counted as referral for fertility preservation. Other post‐intervention survey data were collected to develop future QI initiatives.

### 2.5 Analysis

Continuous variables were compared using Student's *t*‐test for 2 groups. *χ* ^2^ test was used for categorical variables. *p‐*Values <.05 were considered significant.

## 3 RESULTS

### 3.1 Patient characteristics

Post‐intervention survey respondents were older compared to chart‐review cohort but were similar in terms of sex assigned at birth and disease class (Supplemental Table S1). Post‐intervention survey respondents were more likely to meet referral criteria.

### 3.2 Performance following pediatric oncofertility team implementation

**FIGURE 1:**
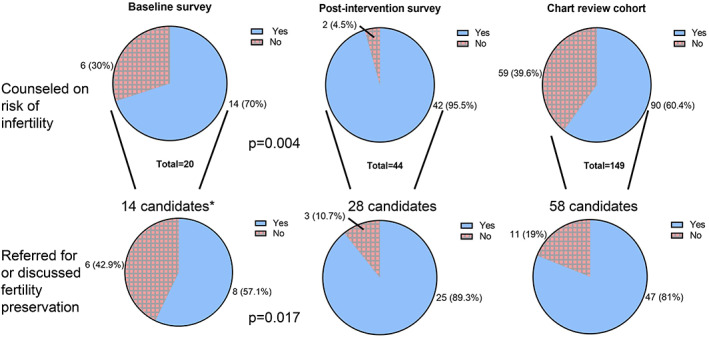
Counseling for the risk of infertility and referral/discussion of fertility preservation procedures prior to and following the implementation of the oncofertility team. *In the baseline survey, the algorithm determining which patients to discuss fertility preservation procedures with had not been instituted. Thus, all 14 patients who received counseling regarding the risks of infertility were considered candidates. Following the implementation of the algorithm, only patients who fit the criteria as outlined in Supplemental Figure S1 were considered candidates. **p‐value between baseline survey and chart review cohort is .407 for counseling and .059 for referral

After pediatric oncofertility team implementation, analysis of post‐intervention survey responses revealed a higher frequency of counseling (95.5% post‐intervention vs. 70% baseline, *p* = .004) and referral (89.3% vs.57.1%, *p* = .017) (Figure 1). A greater proportion of respondents to both surveys recalled counseling than the rate of fertility counseling documented for the chart‐review cohort (60.4%). The rate of referral for fertility preservation in the chart‐review cohort (47/58, 81%) exceeded the baseline rate (8/14, 57.1%, *p* = .059, n.s.). One out of 16 (6.3%) post‐intervention survey respondents and 6/91 patients (6.6%) in the chart‐review cohort who did not meet our criteria elected to pursue FP.

### 3.3 Referral outcomes

Twenty out of 25 (80%) post‐intervention survey respondents who were referred successfully completed FP. All 5 patients who did not complete FP were male. Reported reasons were: not interested (3), inability to produce semen sample (1), hope for better future fertility options (1). Thirty‐eight of 47 (80.9%) referred patients in the chart‐review cohort were documented completing FP. The other nine patients included: four males who were unable to produce a semen sample; two males who were not interested; and two males and one female without documented outcome. None of our patients cited cost as a reason for declining FP.

### 3.4 Differences in FP based on sex assigned at birth

In the chart‐review cohort, 32/35 males meeting referral criteria (91.4%) were referred, compared with 15/23 females meeting criteria (65.2%, *p* = .013). Seven out of eight females meeting criteria who were not referred (87.5%) were premenarchal. Once they were referred, 14/15 referred females completed FP compared to 24/32 referred males (*p* = .14). Among post‐intervention survey respondents, 6/6 referred females completed FP compared with 10/15 referred males.

### 3.5 Inaccurate recall of counseling

Only 15/42 (35.7%) post‐intervention survey respondents recalled their infertility risk level accurately (Table 1). Poor recollection was more pronounced in patients who began therapy before 2019 (6/26, 23.1%). Patients were more likely to overestimate than underestimate their infertility risk. Most post‐intervention survey respondents were unable to recall which therapies they received caused infertility risk (23/44, 52.3%). The most common therapy which was forgotten was a specific alkylating chemotherapy (22), while four respondents forgot receipt of radiation therapy (three respondents forgot both an alkylating chemotherapy and radiation therapy).

**TABLE 1:** Survey respondents recall of their risk of infertility or risk factors

	Year beginning cancer treatment (*n* = number of post‐intervention survey respondents)							
Understanding of fertility risk	2014	2015	2016	2017	2018	2019	2020	Total
Accurate	1	2	0	2	1	8	1	15 (35.7%)
Inaccurate	1	4	4	7	4	7	0	27 (64.3%)
*Overestimate*	*0*	*2*	*2*	*0*	*2*	*3*	*–*	*9*
*Underestimate*	*0*	*0*	*2*	*2*	*0*	*1*	*–*	*5*
*Does not recall level of risk*	*1*	*2*	*0*	*5*	*2*	*3*	*–*	*13*
Total	2	6	4	9	5	15	1	42^a^

^a^ Post‐intervention survey respondents who did not recall receiving counseling (2) were excluded.

## 4 DISCUSSION

We report our outcomes following 7 years of a pediatric oncofertility team improving fertility counseling and referral. A strength of our assessment is that we analyzed a post‐intervention survey plus EMR documentation. While both analyses have limitations (response and recall bias, as well as a lack of standardized timing of survey relative to patient diagnosis in the former; incomplete EMR documentation in the latter), together they provide a more complete picture of our oncofertility outcomes. Both analyses show that following pediatric oncofertility team implementation, our division has largely improved in providing counseling to all patients embarking on gonadotoxic therapy with curative intent and referral for fertility preservation when appropriate. Thus, in an academic pediatric oncology practice, the referral rate can be increased with a pediatric oncofertility team.^13^ This is particularly notable because the effect is seen many years after the initial intervention. We believe that involvement of multiple disciplines has been pivotal for this effect over an extended period. Furthermore, our rate of EMR documentation of FP counseling and referral is in line with what has been reported from another center with defined FP guidelines (60.4%, compared with 63.8% from Salsman et al.)^25^ and is superior to the rate reported from another series by Quinn et al of adolescent and young adult patients at centers where no specific guidelines have been implemented (26%).^26^ While work remains to optimize EMR documentation of FP discussions, the presence of guidelines or a pediatric oncofertility team may increase adherence to this important component of quality care.

Our post‐intervention survey respondents frequently did not retain an accurate understanding of their infertility risk. This understanding was lower in patients who began therapy earlier. Initial counseling regarding infertility risk may be forgotten in the context of an initial consent discussion that may overwhelm patients and parents with the volume and nature of information. We now recommend reinforcing the message at the end of therapy with a written treatment summary. As reported in another cohort,^27^ our patients overestimated their infertility risk. This represents an opportunity to deliver hopeful news that our patients' lives “after cancer” can potentially include conceiving naturally. Furthermore, an incorrect assumption of infertility may increase the risk of unplanned pregnancy.

We also analyzed fertility outcomes between sexes. In other reports, roughly half of adolescent males at risk for infertility attempt sperm banking.^28^ Our males meeting criteria attempted and succeeded at FP at a higher rate (67% post‐intervention survey, 75% chart‐review cohort), demonstrating the impact of our pediatric oncofertility team and the implementation of a standardized referral algorithm. Interestingly, nearly all females who were referred completed FP. While this difference did not reach statistical significance, likely due to relatively low sample size, this suggests a cost–benefit analysis for males and reflects the asymmetry of our algorithm. Because sperm banking is relatively non‐invasive, we offer it to all post‐pubertal boys who will receive any gonadotoxic chemotherapy. For males with low infertility risk, the cost of collection and storage of sperm may be difficult for patients and families to justify. In contrast, we recommend referring only high‐risk females because female FP is more invasive and time‐intensive. These patients and families may find the cost justified by their high infertility risk. Additionally, since the time of our survey, new recommendations have been published by the Pediatric Initiative Network of the Oncofertility Consortium which should be used for infertility risk stratification.^29^

Finally, a surprising number of females in the chart‐review cohort who met referral criteria were not referred. As 7/8 of these girls were premenarchal, this may reflect lingering unfamiliarity with OTC, despite OTC now becoming standard.^30^ Also, the cost of OTC may be prohibitive if it is not combined with another operative procedure that is covered by third‐party payors, such as port placement for chemotherapy administration. While the need for OTC may not be recognized until after initial procedures have been completed, OTC should be considered later if it can be coordinated with another operating room procedure.

There are a number of limitations inherent to our analysis, as discussed above, including the potential for recall and response bias in our post‐intervention surveys and potential incomplete EMR documentation in our chart review cohort. We believe these are mitigated to some extent by our combined approach. Additionally, as the entire field of FP gained greater attention during the timeframe covered here, it is possible that some of the improvement in FP outcomes observed is attributable to factors external to our oncofertility team.

There are a number of exciting directions to be pursued in future studies. We believe our observations justify an effort to replicate our findings at other centers to assess their external validity. Similar tertiary centers could use this framework, in conjunction with guidelines from organizations such as the Pediatric Initiative of the Oncofertility Consortium,^10^, ^31^, ^32^, ^33^ and would be well poised to validate our findings. How a similar framework could be established in settings which have fewer resources readily available, such as availability of OTC and financial support for fertility preservation, is an important goal that several groups are pursuing.^34^, ^35^ In addition to improving patient understanding by reinforcing fertility counseling after diagnosis, further studies to better understand potential differences in FP outcomes for males and females will be of great value. Furthermore, to ensure that FP outcomes are equitably obtained by all patients, it would be intriguing in a future study to consider outcomes stratified by race and ethnicity.^36^ Finally, as novel treatments such as targeted therapies and immunotherapy are integrated into standard care, their impact on fertility needs to be studied to enable appropriate FP counseling of patients receiving these treatments.^37^, ^38^, ^39^

In summary, we implemented a multi‐disciplinary pediatric oncofertility team and developed counseling and referral algorithms with improvement in FP counseling and referral across an extended period of time. While there remains opportunity for continued improvement, our framework can be utilized by other centers.

## AUTHOR CONTRIBUTIONS

**John A Ligon:** Conceptualization (lead); data curation (lead); formal analysis (lead); investigation (lead); methodology (lead); project administration (lead); writing – original draft (lead); writing – review and editing (lead). **Masanori Hayashi:** Conceptualization (supporting); data curation (supporting); formal analysis (supporting); methodology (supporting); writing – review and editing (supporting). **Devon Ciampa:** Conceptualization (supporting); data curation (supporting); investigation (supporting); project administration (supporting); writing – review and editing (supporting). **Cara Kramer:** Conceptualization (supporting); data curation (supporting); project administration (supporting); writing – review and editing (supporting). **Alfredo Guastella:** Project administration (supporting); resources (supporting); writing – review and editing (supporting). **Robert Fuchs:** Data curation (supporting); formal analysis (supporting); investigation (supporting); writing – review and editing (supporting). **Amin Herati:** Methodology (supporting); project administration (supporting); resources (supporting); writing – review and editing (supporting). **Mindy Christianson:** Conceptualization (supporting); investigation (supporting); methodology (supporting); project administration (supporting); resources (supporting); writing – review and editing (supporting). **Allen R Chen:** Conceptualization (lead); investigation (lead); methodology (lead); project administration (lead); resources (lead); writing – original draft (supporting); writing – review and editing (lead).

## CONFLICTS OF INTEREST

The authors have stated explicitly that there are no conflicts of interest in connection with this article.

## ETHICS STATEMENT

Our QI project assessment was approved by the Johns Hopkins Medicine Institutional Review Board, IRB# 00243941, and our project conforms to recognized standards.

## Supporting information


**Supplemental Figure S1:** Flowsheets for fertility counseling and recommendation for referral for fertility preservation procedures. Algorithms for (A) male and (B) female pediatric oncology patients. Low, intermediate, and high risk for infertility were defined in the onco‐fertility policy based on the exposure to alkylating chemotherapy and radiation (Supplemental methods S2). A reference for tanner staging was also included in the onco‐fertility policy. Urgent therapy implies the need to start therapy within 1 week. Emergent therapy implies the need to start therapy within 12 h. GnRH = gonadotropin releasing hormone. OTC, ovarian tissue cryopreservation.

**Supplemental Figure S2:** Consort diagrams for collection and analysis of fertility data from (A) post‐intervention paper survey of pediatric oncology patients who started therapy between 2014 and 2020 and (B) chart review of pediatric oncology patients who started chemotherapy in 2019. JHH, Johns Hopkins Hospital. *Counseling regarding the risk of infertility was provided to both of these patients.

**Supplemental Table S1:** Demographics and disease class for patients responding to the post‐intervention survey and in the confirmatory cohort. *p*‐value for age based on Student's t‐test; *p*‐value for sex assigned at birth, candidacy for fertility preservation, and disease class based on *χ* ^2^ test. BMT, bone marrow transplant.

**Supplemental methods S1:** Oncofertility policy.

**Supplemental methods S2:** Fertility risk stratification for males and females.

**Supplemental methods S3:** Fertility patient questionnaire.

## Data Availability

Data is available upon reasonable request.
