# Supplementary figures and images for: A multidisciplinary pediatric oncofertility team improves fertility preservation and counseling across 7 years
Source: Cancer Rep (Hoboken). 2022 Nov 8;6(2):e1753. doi: 10.1002/cnr2.1753 (PMC9939996; doi:10.1002/cnr2.1753)

A

Male fertility flowsheet

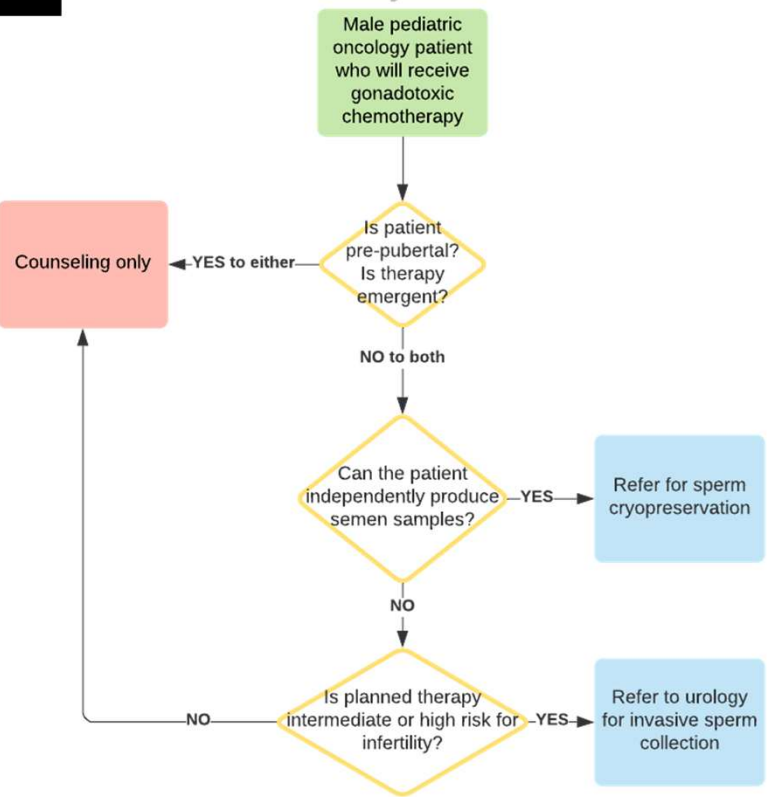

B

Female fertility flowsheet

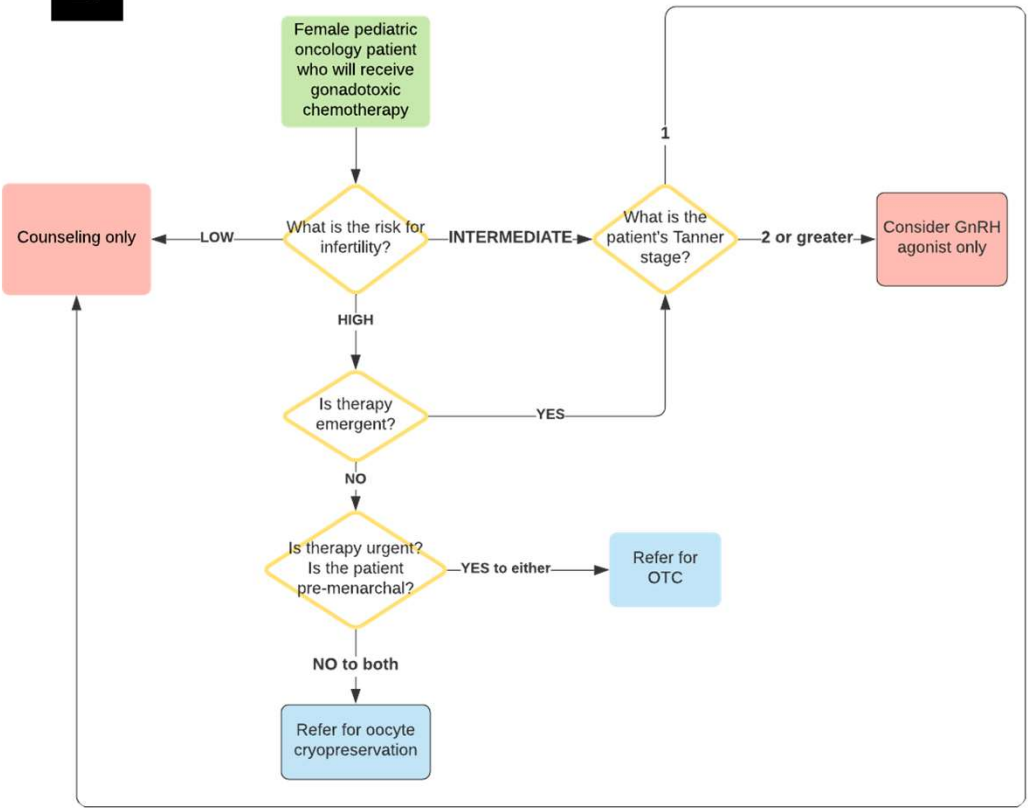

Supplemental Figure S1

Supplement: Supplementary file 1 — Supplemental Figure S1: Flowsheets for fertility counseling and recommendation for referral for fertility preservation procedures. Algorithms for (A) male and (B) female pediatric oncology patients. Low, intermediate, and high risk for infertility were defined in the onco‐fertility policy based on the exposure to alkylating chemotherapy and radiation (Supplemental methods S2). A reference for tanner staging was also included in the onco‐fertility policy. Urgent therapy implies the need to start therapy within 1 week. Emergent therapy implies the need to start therapy within 12 h. GnRH = gonadotropin releasing hormone. OTC, ovarian tissue cryopreservation. [file CNR2-6-e1753-s005.pdf]

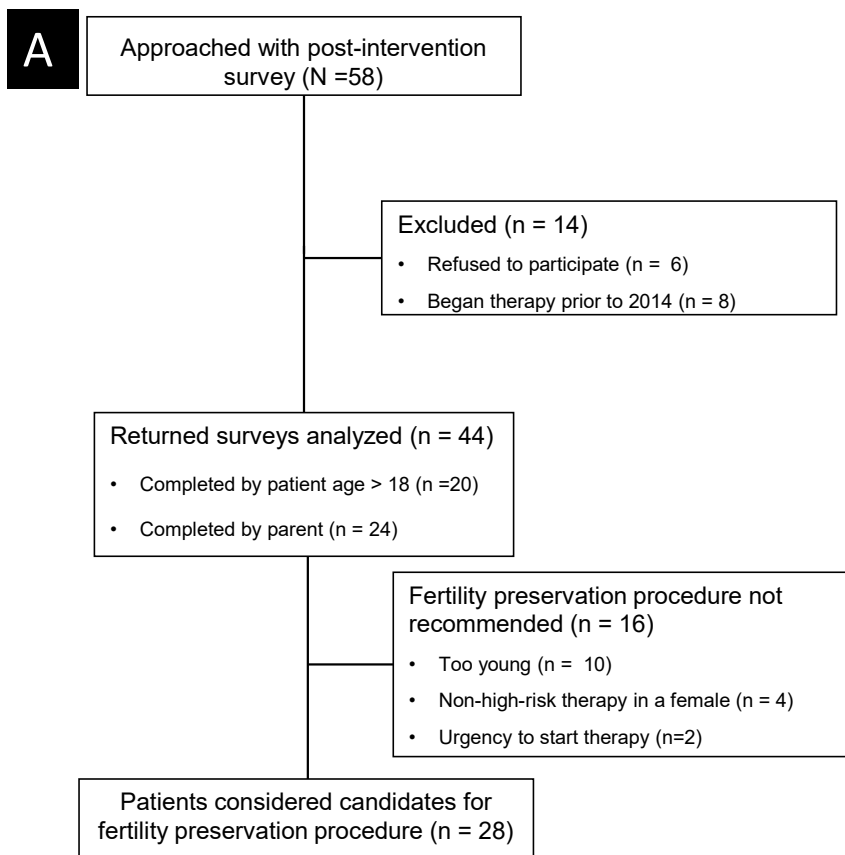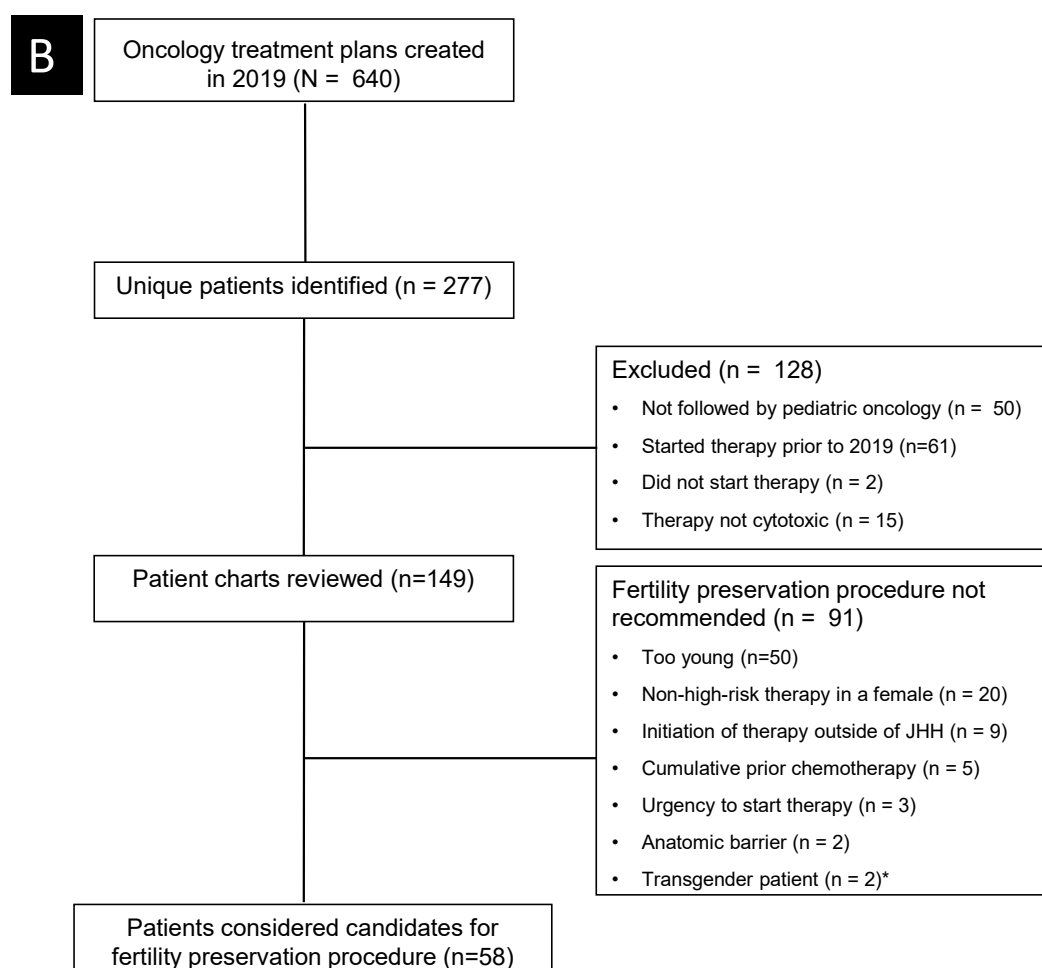

**Supplemental Figure S2**

Supplement: Supplementary file 2 — Supplemental Figure S2: Consort diagrams for collection and analysis of fertility data from (A) post‐intervention paper survey of pediatric oncology patients who started therapy between 2014 and 2020 and (B) chart review of pediatric oncology patients who started chemotherapy in 2019. JHH, Johns Hopkins Hospital. *Counseling regarding the risk of infertility was provided to both of these patients. [file CNR2-6-e1753-s004.pdf]
