# Supplementary material for: A multidisciplinary pediatric oncofertility team improves fertility preservation and counseling across 7 years
Source: Cancer Rep (Hoboken). 2022 Nov 8;6(2):e1753. doi: 10.1002/cnr2.1753 (PMC9939996; doi:10.1002/cnr2.1753)
Supplement: Supplementary file 3 — Supplemental Table S1: Demographics and disease class for patients responding to the post‐intervention survey and in the confirmatory cohort. p‐value for age based on Student's t‐test; p‐value for sex assigned at birth, candidacy for fertility preservation, and disease class based on χ 2 test. BMT, bone marrow transplant. [file CNR2-6-e1753-s006.pdf]

**Supplemental Table S1: Demographics and disease class for patients responding to the post-intervention survey and in the confirmatory cohort.**			
	Post-intervention survey respondents (n=44)	Chart review cohort(n=149)	p-value
**Median age in years at therapy start (range)**	16 (2-24)	11 (0-24)	p=0.0012
**Median months from therapy start to return of survey (range)**	26 (1-70)	-	
**Female (%)**	15 (34.1)	58 (38.9%)	p=.56
**Candidate for fertility preservation (%)**	28 (63.6)	58 (38.9)	P=0.0038
Disease class (%) – Candidates (% of disease class)			p=.20
**Leukemia/lymphoma**	24 (54.5) – 13 (54.2)	56 (37.6) – 18 (32.1)	
**Sarcoma**	11 (25) – 9 (81.8)	36 (24.2) – 20 (55.6)	
**Neuro-oncology**	3 (6.8) – 3 (100)	18 (12.1) – 4 (22.2)	
**Other malignancy**	2 (4.5) – 1 (50)	19 (12.8) – 2 (10.5)	
**Nonmalignant BMT**	4 (9.1) – 2 (50)	19 (12.8) – 14 (73.7)	
**Note: All survey data reflects the patient, regardless of whether the patient or his/her parent responded to the survey**			
