# Supplementary material for: A multidisciplinary pediatric oncofertility team improves fertility preservation and counseling across 7 years
Source: Cancer Rep (Hoboken). 2022 Nov 8;6(2):e1753. doi: 10.1002/cnr2.1753 (PMC9939996; doi:10.1002/cnr2.1753)
Supplement: Supplementary file 4 — Supplemental methods S1: Oncofertility policy. [file CNR2-6-e1753-s003.pdf]

JOHNS HOPKINS MEDICINE THE JOHNS HOPKINS HOSPITAL	The Johns Hopkins Hospital **Children's Center Pediatric Policies, Procedures and Protocols** **Pediatric Oncology**	*Policy Number*	ONC010
		Effective Date	10/05/2021
		Approval Date	N/A
	Subject **Fertility, Counseling and Management in Pediatric Oncology Patients**	*Page*	1 of 6
		Supersedes Date	04/02/2018

Keywords: fertility, infertility, sperm, sperm banking

Table of Contents	Page Number
I. OBJECTIVES	1
II. INDICATIONS FOR USE	1
III. DEFINITIONS	2
IV. RESPONSIBILITY	2
V. ASSESSMENT	3
VI. INTERVENTIONS	4
VII. DOCUMENTATION	6
VIII. SUPPORTIVE INFORMATION	6
IX. SIGNATURES	6
Appendix A: Stratification Guidelines for Female Infertility Risk Assessment	Click Here
Appendix B: Stratification Guidelines for Male Infertility Risk Assessment	Click Here
Appendix C: Alkylators by Disease	Click Here
Appendix D: Oncology Fertility Contact List	Click Here
Appendix E: Male Cryopreservation Options	Click Here
Appendix F: Notary list	Click Here
Appendix G: Cryochoice Mail Order Kit Instructions	Click Here
Appendix H: Cryochoice Account Authorization Form	Click Here
Appendix I: Tanner Staging	Click Here

I. OBJECTIVES

To establish a standard practice of timely fertility counseling and referral of services for pediatric cancer patients who are treated at our institution.

II. INDICATIONS FOR USE

This policy is to be employed for all pediatric oncology patients at Johns Hopkins Children's Center who meet one or more of the following conditions.

A. Any patient with a diagnosis who will require at least one of the following treatments:
   1. Systemic cytotoxic chemotherapy, including but not limited to the gonadotoxic agents listed below, and including stem cell or bone marrow transplant preparative regimens which contain these agents, with or without radiation.
      a. Note: For gender based risk stratification, see appendix.
   2. Radiation therapy in the regions of the gonads, pelvis, abdomen or CNS with the following guidelines to identify particularly high risk patients. Current data available suggests males and females have different sensitivity, and for specifics, please refer to appendix:
      a. Abdominal/pelvic irradiation ≥ 5Gy
      b. Spinal irradiation ≥ 25Gy
      c. Testicular irradiation ≥ 2Gy
      d. Any combination of abdominal/pelvic, spinal, testicular or total body irradiation and alkylating agents.

© Copyright 2021 by The Johns Hopkins Health System Corporation and/or The Johns Hopkins University

3. Surgical procedure(s) that may alter the anatomy or function of the reproductive organs.
4. Any female patients who previously received gonadotoxic therapies.

III. DEFINITIONS

Alkylating agents	Gonadotoxic chemotherapy agents including cyclophosphamide, procarbazine, melphalan, busulfan, platinum agents, ifosfamide, nitrogen mustard, thiotepa.
AMH	Anti-mullerian hormone.
Fertility preservation	Education, counseling, and specialized protocols or procedures to limit the impact of gonadotoxic therapy on future fertility.
FSH	Follicle stimulating hormone.
GnRH	Gonadotropin-releasing hormone.
Gonadotoxic	Damage to the gonadal tissue that impairs function and fertility, such as radiation therapy or cytotoxic chemotherapy.
Impaired fertility	Conception is possible but less likely compared to the normal healthy population.
Infertility	The inability to conceive after 12 months of unprotected sex.
LH	Luteinizing hormone.
Oocyte cryopreservation	Removal and freezing of a woman’s eggs (oocytes), typically reimplanted as an embryo after thawing and fertilization.
Ovarian cryopreservation	Removing and slowly freezing ovarian tissue for future autotransplantation.
Sperm cryopreservation	Freezing sperm cells that can be used in assistive reproduction, typically via intracytoplasmic sperm injection, at a later time.

IV. RESPONSIBILITY

A. Physicians: The primary oncology physicians (attending and fellow) for each patient are responsible for assessing fertility risk and facilitating discussions of fertility preservation options with the other members of the primary team, patient, and family. Specific responsibilites are as follows:
   1. Identify the anticipated use of gonadotoxic therapies, as defined above and further detailed in the appendix, in the patient’s treatment regimen.
   2. Perform a pre-treatment comprehensive physical exam that includes determination and documentation of Tanner stage.
   3. Obtain and review data regarding any previous treatment and any previous utilization of fertility preservation measures for patients who are not newly diagnosed.
   4. Meet with other members of the primary team, including nursing and social work, to discuss the expected fertility risk, determine eligibility for available fertility preservation options and consider recommendations for the patient. The risks and benefits of each option, including the possibility of delaying the initiation of therapy, should be identified.
   5. Discuss any anticipated disease- or treatment-related risk to fertility with the patient and family and provide information regarding available fertility preservation options. This discussion should include counseling on the

© Copyright 2021 by The Johns Hopkins Health System Corporation and/or The Johns Hopkins University

risks and benefits of each option, including any possibility of delaying cancer therapy. Promptly arrange for consultation with other subspecialists (such as surgery, urology, etc) if appropriate and/or requested. **Counseling for onco-fertility should always be provided as an essential piece when discussing about treatment plans and decisions.** The primary oncology fellow will be responsible to document this discussion in the treatment initiation note.

B. Registered Nurse: One or more members of the primary nursing team for each patient, inpatient and/or outpatient members, will be involved in the discussions of infertility risk and fertility preservation and assist in counseling and education on fertility preservation.

1. Nursing will participate in initial meeting(s) with other team members and with patient and family to provide input from the nursing standpoint, discuss fertility risk, eligible preservation options, and recommendations. Nursing should be encouraged to provide any pertinent information regarding the family's knowledge, perspective or preferences regarding fertility preservation to optimize communication and assist in making appropriate recommendations for the patient.
2. Coordinate with social work to facilitate inside or outside referrals as needed and assist in the preparation for preservation procedures.

C. Social Worker: The social worker assigned to each patient will be actively involved in the discussions of infertility risk and fertility preservation and assist in counseling and education on fertility preservation.

1. Provide relevant psychosocial information and assessment to aid in the formulation of recommendation(s) as a primary team prior to initial counseling to patient.
2. Participate in initial meeting with the patient and family to discuss fertility risk and fertility preservation options.
3. Provide psychosocial support and counseling as needed to patients and family members who are coping with the possibility of fertility impairment in addition to a cancer diagnosis.
4. Provide information regarding any possible financial assistance if a fertility preservation option(s) is offered and the patient and family chose to pursue one of these options.
5. Facilitate inside and outside referrals.

D. Onco-fertility team: an interdisciplinary team of faculty, fellows, nurses and social workers who participate in the Onco-fertility Quality Improvement (QI) initiative. The team will accept the following responsibilities:

1. Upon consultation, members of this team will serve as a liaison within the pediatric oncology division to facilitate referrals to reproductive endocrinology, provide additional counseling, or connect patients or providers with additional resources.
2. Designate an on-call physician, nurse, and social worker to answer any emergent questions.
3. Meet regularly to review employment of the policy and make any adjustments as needed.

E. Reproductive Endocrinology

1. Reproductive Endrocrinology will be available as consultants to provide expertise to the primary team and counsel and inform patients on risks and benefits related to fertility preservation methods when necessary.
2. Reproductive endocrinology will join the Pediatric oncology onco-fertility team to provide input and expertise to continuously evaluate and improve the policy.

V. <u>ASSESSMENT</u>

"Risk assessment" mentioned in the following section refers to "permanently gonadotoxic therapy." Any kind of chemotherapy can be cytotoxic to mature spermatozoa, and cause temporary azospermia. Therefore, assessment and intervention should be ideally performed prior to the administration of ANY CHEMOTHERAPY.

A. The primary oncology physicians will determine the likelihood of fertility impairment as noted above. Gender specific flowsheets can be found in the appendices.

© Copyright 2021 by The Johns Hopkins Health System Corporation and/or The Johns Hopkins University

B. A comprehensive physical exam shall be performed by the treating physicians, after which the patient's Tanner stage will be determined. Tanner Stage III will be the threshold for defining a patient as pubertal.

C. An additional component of the physician assessment will be urgency of initiation of disease-directed therapy in relation to the timing of appropriate fertility preservation options. Many fertility preservation methods involve an at least modest delay in therapy. The risk of delay of therapy will be weighed against the benefit of fertility preservation, the likelihood of success with the selected option, and the risks and invasiveness of the procedure. If any of these items is unclear, the onco-fertility team should be consulted.

D. Long term survivors should be assessed for late fertility risk or, in females, premature ovarian failure. Assessment should include a symptom inventory, particularly for female patients, hormonal studies including FSH, LH, inhibin, testosterone, AMH and semen analysis. For qualifying female survivors (those who received "high" or "intermediate" risk therapies according to the flowsheet), early oocyte cryopreservation may be recommended. Male survivors determined to be fertile and interested in sperm banking should wait at least one and ideally two years from the completion of therapy.

VI. INTERVENTIONS

A. Males

1. Males with a new diagnosis who are capable of providing their own semen samples and will be receiving any gonadotoxic therapy will be promptly referred for sperm cryopreservation (see Appendices for gender-specific information). For peri-pubertal patients with no prior experience of masturbation, attempts of sperm collection is not recommended. Sperm collection should be performed prior to receiving any therapy. The ideal recommendation is three samples each at at least 24 hours apart. For those requiring very urgent therapy, fewer samples or shorter duration between samples can and should be considered. The same procedure can be followed for previously diagnosed patients who have not received gonadotoxic therapy or patients who have had an extended interval (1-2yr) since their therapy and did not previously bank sperm.

2. For pubertal or post-pubertal patients who cannot provide their own semen samples, invasive collection of sperm by urology for cryopreservation can be utilized if patient will be receiving **intermediate or high risk gonadotoxic therapy (Appendix B)**. When invasive collection is necessary, the oncology provider for the patient should contact Dr. Amine Herati (aherati1@jhmi.edu). Briefly, those options may include:

    a. **Testicular sperm extraction (TESE)**: Outpatient procedure under general anesthesia during which a small amount of testicular tissue is removed and viable sperm stored This may be used in cancer survivors who have non-obstructive azospermia, but success rates are not as high.

    b. **Electroejaculation**: electric probe placed under general anesthesia.

    c. **Epidydimal sperm aspiration**: direct removal of sperm from tubule.

3. Follow-up

    a. Patients who have had gonadotoxic therapy (regardless of risk of permanent infertility) often have azoospermia and or chromosomal abnormalities for up to 24 months after completion of therapy. Re-evaluation of sperm should be performed at 24 months off therapy for sperm number and motility at the facility where sperm is stored, or at reproductive endocrinology (order available in Epic) in cases where sperm was not cryopreserved. Live viable sperm, even with decreased count and motility, is preferred over cryopreserved and thawed sperm for IVF. Therefore, with confirmation of live sperm, patients may consider discarding their cryopreserved sperm samples. This decision should also be made with strong consideration of relapse risk, and where the risk is high, cryopreservation should be continued.

B. Females

1. Oocyte cryopreservation

© Copyright 2021 by The Johns Hopkins Health System Corporation and/or The Johns Hopkins University

a. For post menarche patients who can potentially delay therapy for 2 weeks with acceptable risk, oocyte cryopreservation can be the first choice for fertility preservation.
b. If a patient seems to be a good candidate for oocyte cryopreservation, a reproductive endocrinology consult is warranted to provide additional information to the family and coordinate treatment.

2. Ovarian cryopreservation (Off study)
    a. For patients who do not qualify for the ovarian cryopreservation study, but are still considered to be at high risk of loss of fertility and would benefit from ovarian cryopreservation, a prompt referral to reproductive endocrinology will be made.
    b. The risks of fertility loss should be assessed appropriately due to financial burden and invasiveness and ovarian cryopreservation should be recommended when there is a high risk for fertility loss. If inquired, it is still important to provide necessary information and referrals for ovarian cryopreservation even when fertility loss risk is not high, but the primary team should offer appropriate counseling and risk assessment from an oncologic standpoint.
    c. For patients who are appropriate candidates and are interested in ovarian tissue cryopreservation outside the study, contact Dr. Mindy Christianson (Dr. Christianson coordinates cryopreservation with the lab).
    d. A transabdominal pelvic ultrasound should be ordered prior to harvest to assess normal anatomy, ovarian size and location.
3. Ovarian cryopreservation study is currently closed.
4. GnRH analog
    a. GnRH analogues such as Lupron have been used with mixed data for patients who are planning to undergo fertility altering therapy.While GnRH analogs remain controversial regarding their benefit from a fertility preservation perspective, they offer other benefits such as suppression of menses.
    b. Recommended Lupron (leuprolide acetate for depot suspension) dosing is 3.75 mg monthly or 11.25 mg for 3 months.
    c. Starting dose will be monthly dose, which will be followed by every 3 month dose.
    d. Optimally, Lupron should be administered 7-10 days prior to gonadotoxic treatment. If a patient needs to start chemotherapy right away, it is fine to administer Lupron closer to treatment.
        i. All post-menarchal patients who will be receiving intermediate to high risk gonadotoxic therapy should be offered Lupron. Additionally, girls in early puberty (e.g. breast development) should be offered Lupron as ovaries are active at this time. There is no benefit to offering Lupron to pre-pubertal girls as their ovaries are not yet active and already suppressed.
        ii. Initially, patients should anticipate a "flare" which often results in menstrual bleeding. After this initial bleed, the majority of patients will become amennorrheic.
        iii. Side effects of Lupron can include anti-estrogen effects such as hot flashes. If these are debilitating to patients, estrogen add-back therapy can be used.
            - Gynecology should be consulted for additional dosing guidelines on this.
            - Typical estrogen add-back therapy may include Estrace 1 mg daily (if treatment 4 months or less) or an oral contraceptive pill with 30 mcg of estradiol taken continuously with no placebo pills (if treatment expected to exceed 4 months).
5. Oophoropexy
    a. When the patient is receiving direct pelvic or abdominal radiation, surgical transposition of the ovaries should be attempted to avoid direct radiation.
6. Monitoring

© Copyright 2021 by The Johns Hopkins Health System Corporation and/or The Johns Hopkins University

a. For post pubertal patients, measurement of pretreatment hormonal status is recommended for future use. This includes FSH, Inhibin B, AMH and ovarian measurement by ultrasound.

7. Follow-up
   a. Reproductive endocrionology consult should be considered for patients who have not resumed periods 12 months after completion of therapy, or for patients who were considered high risk for fertility impairment or premature ovarian failure.

VII. DOCUMENTATION

The discussion of therapy or disease-related infertility risk and fertility preservation options should be documented in the electronic medical record as part of the pre-treatment evaluation by the physician, and initial social work evaluation when applicable.

VIII. SUPPORTIVE INFORMATION

References:

1. Fertile Hope (Livestrong): http://www.fertilehope.org/
2. ASCO Survivorship Guidelines for Fertility Preservation
3. NCCN Adolescent & Young Adult Guidelines
4. The Oncofertility Consortium: http://oncofertility.northwestern.edu
5. COG Long term follow up guidelines

Reviewed by:

- Pediatric Oncology
- Pediatric Oncology Nursing

IX. SIGNATURES

Electronic Signature(s)	Date
Dawn Luzetsky<br>Associate CNO Pediatric Nursing	10/05/2021
Michael Barbato<br>Pediatric Oncology Fellow	09/17/2021

© Copyright 2021 by The Johns Hopkins Health System Corporation and/or The Johns Hopkins University
