# Supplementary material for: A multidisciplinary pediatric oncofertility team improves fertility preservation and counseling across 7 years
Source: Cancer Rep (Hoboken). 2022 Nov 8;6(2):e1753. doi: 10.1002/cnr2.1753 (PMC9939996; doi:10.1002/cnr2.1753)
Supplement: Supplementary file 5 — Supplemental methods S2: Fertility risk stratification for males and females. [file CNR2-6-e1753-s001.pdf]

Stratification guidelines for male infertility risk assessment

A. High Risk
   1. Moderate to high dose alkylators
      a. MOPP (mechlorethamine, VCR, procarbazine, prednisone) for 3 or cycles
      b. Busulfan >600mg/m2
      c. Cytoxan >7.5gm/m2 or as part of BMT conditioning
      d. Ifosfamide >60gm/m2
   2. Total Body Irradiation
   3. Testicular Irradiation >2Gy
   4. Any alkylator combined with any testicular irradiation

B. Intermediate Risk
   1. Any other multiple alkylator-containing treatment regimens
   2. Any other treatment regimens which include gonadal exposure to irradiation

Stratification guidelines for female infertility risk assessment

A. High Risk
   1. Moderate to high dose alkylators
      a. Cytoxan >7.5gm/m2 or as part of BMT conditioning
      b. High dose Carboplatin therapy requiring stem cell rescue
      c. Stem cell transplant therapy with high dose chemotherapy or TBI as preparation
      d. Alkylating score ≥ 3rd tertile according to attached chart
   2. Whole abdominal or pelvic radiation.
      ≥ 15 Gy in pre-pubertal girls
      ≥ 10 Gy in post-pubertal girls
   4. Any alkylator combined with any pelvic irradiation

B. Intermediate Risk
   1. Whole abdominal or pelvic radiation
      10-<15 Gy in prepubertal girls
      5-<10 Gy in postpubertal girls
      Spinal radiation >/= 25 Gy
   2. Alkylating score = 2rd tertile according to attached chart

C. Alkylator score chart

	Cumulative dose by tertile		
	First	Second	Third
BCNU, mg/m2	1-300	301-529	530-
Busulfan, mg/m2	1-317	318-509	510-
CCNU, mg/m2	1-361	362-610	611-
Chlorambucil, mg/m2	1-165	166-634	635-
Parental Cyclophosphamide, mg/m2	1-3704	3705-9200	9200-
Oral cyclophosphamide, m2	1-4722	4723-10636	10637-
Ifosfamide mg/m2	1-16771	16772-55758	55759-
Melphalan, mg/m2	1-39	40-137	138-574
Nitrogen Mustard, mg/m2	1-44	45-64	65-
Procarbazine, mg/m2	1-4200	4201-7000	7001-
Thiotepa, mg/m2	1-77	78-220	221-
Thiotepa, intrathecal mg/m2	1-80	81-320	321-
