# Supplementary material for: A multidisciplinary pediatric oncofertility team improves fertility preservation and counseling across 7 years
Source: Cancer Rep (Hoboken). 2022 Nov 8;6(2):e1753. doi: 10.1002/cnr2.1753 (PMC9939996; doi:10.1002/cnr2.1753)
Supplement: Supplementary file 6 — Supplemental methods S3: Fertility patient questionnaire. [file CNR2-6-e1753-s002.pdf]

Onco-fertility care quality improvement project

We are trying to improve our practice of counseling our patients about risks their disease and its treatment may pose to their fertility. To assess how well we are counseling our patients, we are conducting a questionnaire on all patients who have received care at pediatric oncology at Johns Hopkins (regardless of diagnosis or fertility risk). We would greatly appreciate your participation by answering the following brief questionnaire.

Information obtained by this questionnaire will be used only for quality improvement purposes after de-identification of personal information.

Parents should answer on behalf of their child unless the patient is >18yo. Please answer with information about the patient.

Patient demographic and disease information

1. Please list the patient's name and date of birth. This information will be used to link questionnaire answers to medical records for fertility related medical information and to avoid duplicate requests to answer the questionnaire.

   Name ( )

   Date of birth (or age) ( )

2. What was the oncologic diagnosis? Or if the patient was seen in pediatric oncology for a stem cell transplant, what was the diagnosis for which the transplant was performed?

Diagnosis ( )

Date of Diagnosis ( )

3. Based on information about fertility from your care team, what is your understanding of the risk of fertility loss from therapy?

   ☐ Unlikely (<30%)
   ☐ Moderate (30-60%)
   ☐ High (60-90%)
   ☐ Very high (90%-100%)
   ☐ I think I received some form of information but I don't recall these details.
   ☐ I don't think I received any information about likelihood of fertility loss from therapy.

4. Please check the boxes to identify which, if any, of the following medications or therapies the patient received.

   ☐Cyclophosphamide/Cytoxan ☐Ifosfamide ☐Cisplatin ☐Carboplatin
   ☐Melphalan ☐Procarbazine ☐Thiotepa ☐BCNU/Carmustine
   ☐Radiation to whole body ☐Radiation to testes or pelvic area
   ☐Radiation to abdomen ☐Radiation to lower spine

5. Did the patient perform any of the fertility preservation measures listed below?

   Males
   ☐Sperm banking at Shady Grove ☐Sperm banking utilizing CryoChoice kit
   ☐Sperm banking via another method
   ☐testicular sperm extraction ☐Other methods (please specify:)
   Females
   ☐Oocyte cryopreservation (egg freezing) ☐Embryo cryopreservation
   ☐ovarian cryopreservation ☐luprolide/Lupron
   ☐Surgical "tacking" of ovaries before radiation

☐Other methods (please specify:)

6. When were these measures performed?

   ☐Before any therapy started
   Before therapy for relapsed or progressive disease
   ☐During therapy
   ☐After therapy
   ☐Did not perform any

7. If not, what was the reason you did not pursue fertility preservation measures?

   ☐It was unnecessary due to low risk of treatment related fertility loss
   ☐The disease was progressing too fast or was too severe
   ☐Too young to pursue any measures
   ☐I don't know because no one talked about this.
   Financial concerns
   ☐Other reasons. Please specify below.

Thank you very much for your time. We appreciate your valuable input, and through this project, we are aiming to improve the care we provide in onco-fertility.

If there are any questions regarding this questionnaire, please contact

John Ligon, MD jligon1@jhmi.edu
